# miR-320d Is Associated with Reduced Nasopharyngeal Carcinoma Progression, Potentially through the NF-κB/IL-8 Axis-Mediated Inhibition of Neutrophil Extracellular Trap Formation

**DOI:** 10.32604/or.2026.081869

**Published:** 2026-07-16

**Authors:** Liu Liu, Jie Liu, Shuangchen Ning, Jin Wang, Yingchun He

**Affiliations:** 1 The First Hospital of Hunan University of Chinese Medicine, Changsha, China; 2 School of Medicine, Hunan University of Chinese Medicine, Changsha, China; 3 Changsha Hospital for Maternal & Child Health Care Affiliated to Hunan Normal University, Changsha, China

**Keywords:** Nasopharyngeal carcinoma, miR-320d, neutrophil extracellular traps, tumor microenvironment, NF-κB/IL-8 axis

## Abstract

**Objectives:** Nasopharyngeal carcinoma (NPC) is an aggressive head and neck malignancy in which post-treatment recurrence and distant metastasis remain major contributors to poor clinical outcomes. Although microRNAs are important post-transcriptional regulators of tumor progression, the role of miR-320d in NPC remains incompletely understood. This study evaluated the biological role of miR-320d and explored whether it is involved in regulating neutrophil extracellular trap (NET) formation through the nuclear factor kappa-B (NF-κB)/interleukin-8 (IL-8) signaling axis. **Methods:** miR-320d was overexpressed in NPC cell lines S18 and 5-8F, and cell viability, migration, and invasion were evaluated. Integrated transcriptomic and proteomic analyses were performed to identify miR-320d-regulated genes, proteins, and pathways. Western blotting, immunohistochemistry, and immunofluorescence analyses were used to validate NET-associated proteins, including glycoprotein Ib platelet subunit alpha (GP1BA), histone deacetylase 10 (HDAC10), and fibrinogen gamma chain (FGG), as well as the expression of NET formation markers, including peptidyl arginine deiminase 4 (PADI4), myeloperoxidase (MPO), and neutrophil elastase (NE); and key components of the NF-κB/IL-8 axis. **Results:** miR-320d overexpression significantly inhibited the viability, migration, and invasion of both S18 and 5-8F cells. Multi-omics analyses indicated that miR-320d-regulated molecules were mainly enriched in NET-related pathways. Consistently, miR-320d reduced the expression of GP1BA, HDAC10, FGG, PADI4, MPO, and NE. Mechanistically, miR-320d suppressed NF-κB signaling, as shown by decreased phosphorylated NF-κB (p-NF-κB) and total NF-κB levels, and reduced IL-8 secretion in NPC cells. **Conclusion:** miR-320d may suppress NPC progression, at least in part, by attenuating NF-κB/IL-8-associated NET formation. These findings suggest that the miR-320d/NF-κB/IL-8/NET regulatory axis may participate in NPC progression and may warrant further translational investigation.

## 1 Introduction

Nasopharyngeal carcinoma (NPC) is a highly aggressive head and neck malignancy with a distinct geographic distribution, showing particularly elevated incidence rates in East and Southeast Asia [1]. Although the combined use of radiotherapy and chemotherapy has significantly enhanced the survival rates of NPC patients, a considerable number of patients are still diagnosed at a locoregionally advanced stage of the disease, largely because early symptoms are insidious and nonspecific [2]. Moreover, recurrence and distant metastasis after treatment remain the leading causes of death in NPC patients [3]. Therefore, elucidating the molecular mechanisms underlying NPC progression and identifying effective therapeutic targets are of considerable clinical importance.

MicroRNAs (miRNAs) are endogenous posttranscriptional regulators of gene expression that play critical roles in tumor initiation, progression, and therapeutic response. Increasing evidence indicates that miRNAs have considerable potential as diagnostic, prognostic, and therapeutic biomarkers in human cancers [4]. In recent years, miRNA-based therapeutic strategies have received significant attention from researchers globally, as restoration of tumor-suppressive miRNAs or inhibition of oncogenic miRNAs was found to improve treatment response and clinical outcomes [5]. miR-320d, a member of the miR-320 family, is significantly downregulated in cisplatin-resistant ovarian cancer [6]. Moreover, it exerts tumor-suppressive effects in several malignancies, including breast and colorectal cancers, and its decreased expression is associated with poor prognosis [7,8]. miR-320d has also been shown to inhibit tumor progression by targeting key oncogenic molecules, such as METTL3, FoxM1, and TUSC3, in different tumor types [9,10,11]. In NPC, emerging evidence indicates that miR-320d may be modulated by long noncoding RNAs within ceRNA networks, potentially influencing key processes such as epithelial-mesenchymal transition (EMT) [12]. However, the precise biological role of miR-320d and its underlying molecular mechanisms in NPC remain largely unclear.

As part of our preliminary work in the present study, an integrated analysis of differentially expressed genes (DEGs) and proteins between the miR-NC overexpression (oe-miR-NC) and miR-320d overexpression (oe-miR-320d) groups in NPC cells revealed a close relationship between miR-320d-regulated molecules and the neutrophil extracellular trap (NET) pathway.

NPC is strongly associated with Epstein-Barr virus (EBV) infection, which helps shape a distinctive inflammatory tumor microenvironment. This EBV-related microenvironment is characterized by extensive immune cell infiltration and aberrant cytokine and chemokine signaling, which are intricately linked with tumor progression and immune escape [13,14]. NETs are extracellular DNA-protein networks released by activated neutrophils and are now considered important contributors to tumor growth, invasion, metastasis, and immune remodeling within the tumor microenvironment [15,16,17]. Recent evidence further suggests that targeting NET formation may enhance the efficacy of immunotherapy in EBV-associated NPC [18]. Rather than occurring as a passive cellular event, NET formation proceeds as a highly regulated process controlled by multiple inflammatory and stress-related signaling pathways. Thus, interventions at critical molecular nodes governing NET formation could be a promising strategy to block tumor metastasis and immune escape. miRNAs have also been implicated in the regulation of NET formation and cancer-associated inflammatory responses [19]. Nevertheless, it remains unclear whether miR-320d modulates tumor cell-derived chemokine secretion and thereby influences NET formation in NPC.

The objective of this study was to determine the biological role of miR-320d in NPC to investigate the mechanism by which miR-320d regulates NPC progression. Specifically, we aimed to evaluate whether miR-320d suppresses NPC cell viability, migration, and invasion, and to examine whether this effect is associated with inhibition of neutrophil extracellular trap (NET) formation through the NF-κB/IL-8 signaling axis.

## 2 Materials and Methods

### 2.1 Cell Lines and Cell Culture

The human NPC cell lines S18 (Beijing, China; catalog number: BNCC-001) and 5-8F (Beijing, China; catalog number: BNCC-341932) were purchased from Be Na Culture Collection. Short tandem repeat (STR) profiling was used to authenticate all cell lines, and mycoplasma contamination was excluded before use. Cells were cultured in RPMI-1640 medium (Gibco, Grand Island, NY, USA; catalog number: 11875-093) supplemented with 10% heat-inactivated fetal bovine serum (FBS; Gibco; catalog number: 26140-079), 100 U/mL penicillin (Gibco; catalog number: 15140-122), and 100 μg/mL streptomycin (Gibco; catalog number: 15140-122), and maintained at 37°C in a humidified incubator with 5% CO2. Routine passaging was performed with 0.05% trypsin/0.02% EDTA solution (Gibco; catalog number: 25300-054).

### 2.2 Lentiviral Transduction and Generation of Stable Cell Lines

To establish NPC cell models with stable miR-320d overexpression, logarithmically growing S18 and 5-8F cells were plated in 6-well plates at 1 × 10^5^ cells per well and transduced when confluence reached approximately 50%. Lentiviral vectors carrying either a negative control sequence (oe-miR-NC) or miR-320d overexpression construct (oe-miR-320d) were synthesized and provided by GeneChem Co., Ltd. (Shanghai, China; catalog number: CV622). The lentiviral vector backbone used in this study was CV622, which contains a neomycin-resistance selectable marker rather than a puromycin-resistance cassette. This vector enable stable expression of the miR-320d and negative control sequences in mammalian cells. The sequences of both constructs were verified by Sanger sequencing to confirm the correct insertion of miR-320d or negative control sequences. The multiplicity of infection (MOI) was optimized in preliminary experiments and set at 50 MOI based on viral titer and cell number. The viral titer was determined by end-point dilution assay and calculated as infectious units per milliliter (IU/mL). Lentiviral particles were added directly to the culture medium, which was replaced with fresh complete medium 24 h after infection.

Stable cell populations were selected beginning 48 h after infection using G418 (Beyotime Biotechnology, Shanghai, China; catalog number: ST081) at a final concentration of 600 μg/mL. This concentration was determined in preliminary selection experiments to ensure efficient elimination of uninfected cells while maintaining the viability of resistant transduced cells. Selection medium was refreshed every 2–3 days for approximately 2–3 weeks until all uninfected control cells had died and resistant clones remained in the infected groups. The established stable cell lines were named oe-miR-NC and oe-miR-320d and subsequently expanded for downstream experiments. Three independent biological replicates were included for statistical analysis to ensure the reproducibility and robustness of the results. After stable cell-line generation, miR-320d overexpression was confirmed again by quantitative real-time polymerase chain reaction (qRT-PCR) before subsequent functional assays were performed, thereby verifying the efficiency and stability of transgene expression.

### 2.3 qRT-PCR

miRNA was isolated from logarithmically growing cells using a miRNA extraction protocol according to the manufacturer’s instructions. For complementary DNA (cDNA) synthesis, 1 μg of miRNA was used for each reverse-transcription reaction. cDNA was synthesized in a 20 μL reaction system using the NovoScript miRNA First-Strand cDNA Synthesis and SYBR qPCR Kit (Novoprotein Scientific Inc., China; catalog number: 05243903), which is specifically designed for miRNA reverse transcription and quantitative PCR. The reverse-transcription mixture contained miRNA template, 2× miRNA RT Reaction Mix, NovoScript miRNA RT Enzyme Mix, and RNase-free water. Reverse transcription was performed at 39°C for 60 min, followed by enzyme inactivation at 85°C for 5 min.

Quantitative PCR (qPCR) was carried out using 2× miRNA SYBR qPCR SuperMix from the same NovoScript miRNA First-Strand cDNA Synthesis and SYBR qPCR Kit on a StepOnePlus Real-Time PCR System (Applied Biosystems, Thermo Fisher Scientific, Foster City, CA, USA). Each qPCR reaction was prepared in a final volume of 20 μL, containing 10 μL of 2× miRNA SYBR qPCR SuperMix, 1 μL of forward primer, 1 μL of reverse primer, 2 μL of cDNA template, and 6 μL of RNase-free water, and all assays were run in technical triplicate. The primer sequences were as follows: miR-320d forward, 5′-AGCTGGACAAAAGCTGGGTTG-3′ and reverse, 5′-TGGAACGCTTCACGAATTTGCG-3′; U6 forward, 5′-AGAGAAGATTAGCATGGCCCCTG-3′ and reverse, 5′-ATCCAGTGCAGGGTCCGAGG-3′. Each experiment included three independent biological replicates, and no-template controls were incorporated to monitor possible contamination. Cycling was performed with an initial denaturation step at 95°C for 1 min, followed by 40 cycles of 95°C for 5 s and 60°C for 30 s. Relative expression was determined by the 2^−ΔΔCt^ method using U6 as the endogenous control.

### 2.4 Cell Counting Kit-8 (CCK-8) Assay

Stable oe-miR-NC and oe-miR-320d S18 and 5-8F cells were collected and plated in 96-well plates at a density of 3 × 10^3^ cells/well in 100 μL of complete medium. Complete medium consisted of RPMI-1640 medium (Gibco; catalog number: 11875-093) supplemented with 10% FBS (Gibco; catalog number: 26140-079), 100 U/mL penicillin (Gibco; catalog number: 15140-122), and 100 μg/mL streptomycin (Gibco; catalog number: 15140-122). After incubation for 0, 24, and 48 h (the 72 h time point was not included due to potential overconfluence that could affect assay accuracy), 10 μL of CCK-8 reagent (Solarbio, Beijing, China; catalog number: CA1210) was dispensed into each well and incubated for 2 h protected from light. Blank wells containing medium plus CCK-8 reagent alone were included as background controls. Absorbance was measured at 450 nm using a microplate reader (Bio-Rad Laboratories, Hercules, CA, USA; Model 680), and the blank readings were subtracted from the corresponding absorbance values before analysis.

### 2.5 Wound Healing Assay

Stable oe-miR-NC and oe-miR-320d S18 and 5-8F cells were plated in 6-well plates at 1 × 10^5^ cells per well and grown to 95%–98% confluence. A linear scratch was introduced across the monolayer with a sterile 200 μL pipette tip. Detached cells were removed by two gentle PBS washes, after which the remaining cells were maintained in serum-free RPMI-1640 basal medium without FBS throughout the assay. Wound images were acquired at 0 and 24 h using an inverted microscope (Olympus Corporation, Tokyo, Japan; Olympus IX73). The wound area was measured with ImageJ software (Version 1.53c, National Institutes of Health [NIH], Bethesda, MD, USA), and the wound-closure percentage was calculated.

### 2.6 Transwell Invasion Assay

Stable oe-miR-NC and oe-miR-320d S18 and 5-8F cells were harvested and suspended in serum-free medium. Transwell chambers with 8-μm pore size membranes were used, and the upper chambers were precoated with Matrigel (Corning Inc., Corning, NY, USA; catalog number: 354234) diluted 1:8 (initial concentration: 1 mg/mL protein content) and incubated at 37°C for 2 h to allow solidification. Subsequently, 2 × 10^4^ cells in 200 μL serum-free medium were seeded into the upper chamber, while 600 μL of complete medium containing 10% FBS was placed in the lower chamber as a chemoattractant. After incubation for 24 h, non-invading cells remaining on the upper membrane surface were carefully removed with a cotton swab. Cells that had invaded to the lower surface were fixed with 4% paraformaldehyde (Sigma-Aldrich, St. Louis, MO, USA; catalog number: 158127) for 15 min and stained with 0.1% crystal violet (Sigma-Aldrich, catalog number: 190705) for 20 min. After washing with PBS, invaded cells were photographed and counted in five randomly selected fields under an inverted microscope (Leica Microsystems, Shanghai, China; Model: DMI4000B). ImageJ software (Version 1.53e; NIH) was used for quantitative analysis. The average count from five fields was used to represent the invasion level for each well, and data from three independent biological replicates were averaged for statistical analysis.

### 2.7 Transcriptomic and Proteomic Profiling

S18 cells with stable oe-miR-NC or oe-miR-320d expression were harvested for transcriptomic and proteomic analyses. The analysis was performed in S18 cells because this line is well characterized and relevant to NPC; although future validation in additional cell lines will be needed. Transcriptomic and proteomic analyses were conducted using the same batch of cell samples. For each biological replicate, parallel aliquots from the same cell harvest were used for RNA and protein extraction. Each group contained three independent biological replicates (*n* = 3). All analyses were completed by a commercial service provider (BioProfile Biotechnology Co., Ltd., Shanghai, China).

For transcriptomic profiling, total RNA was isolated using TRlzol reagent. A total of 1 μg RNA was used for each reaction, and RNA integrity was evaluated on an Agilent 2100 Bioanalyzer (Agilent Technologies, Santa Clara, CA, USA). All samples showed an RNA integrity number > 8.0. Strand-specific mRNA libraries were constructed with the Illumina TruSeq Stranded mRNA Library Prep Kit (Illumina, San Diego, CA, USA; catalog number: RS-122-2101) and sequenced on the Illumina NovaSeq 6000 platform (Illumina, Model: 20012850) to generate paired-end 150-bp reads. Raw reads were quality-checked with FastQC (Version 0.11.9, Babraham Bioinformatics, Cambridge, UK), and Trimmomatic (Version 0.39; University of Maryland, USA) was used to remove adapter sequences and low-quality reads. Clean reads were mapped to the human reference genome (GRCh38) with STAR (Version 2.7.5a; Cold Spring Harbor Laboratory, Cold Spring Harbor, NY, USA), and featureCounts was applied to quantify gene expression. Differential expression was analyzed with the DESeq2 package (Version 1.30.0; EMBL, Heidelberg, Germany), and *p*-values were corrected by the Benjamini–Hochberg method. DEGs were defined using thresholds of |log_2_ fold change (FC)| ≥ 1 and false discovery rate (FDR) < 0.05.

For proteomic profiling, total protein was isolated with RIPA lysis buffer (Beyotime Biotechnology; catalog number: P0013B) supplemented with protease inhibitors (Beyotime Biotechnology; catalog number: P1005), Extraction was carried out on ice for 30 min with intermittent vortexing. Protein concentration was measured using a bicinchoninic acid protein assay kit (Beyotime Biotechnology; catalog number: P0010). Protein samples were reduced with dithiothreitol (Sigma-Aldrich; catalog number: D0632), alkylated with iodoacetamide (Sigma-Aldrich; catalog number: I1149), and digested with trypsin (Promega, Madison, WI, USA; catalog number: V5111) at an enzyme-to-protein ratio of 1:50. Peptides were separated on a nano-liquid chromatography system (Thermo Fisher Scientific, Bremen, Germany; Model: Easy-nLC 1200), using 0.1% formic acid in water (Sigma-Aldrich, catalog number: F0507) as mobile phase A and 85% acetonitrile (Merck, Darmstadt, Germany; catalog number: 100017) as mobile phase B.

The eluted peptides were analyzed with a Q Exactive HF-X mass spectrometer (Thermo Fisher Scientific, catalog number: 072510) operated in data-dependent acquisition mode. Major mass spectrometry (MS) settings included a 70,000 resolution at m/z 200 for MS1 scans, a scan range of 350–1500 m/z, and a target ion population of 1e5. MS2 acquisition was performed at a resolution of 17,500 at m/z 200. Raw MS data were processed in Proteome Discoverer (Version 2.4; Thermo Fisher Scientific) and searched against the UniProt human protein database (UniProt release 2020_03; UniProt, Switzerland; available at: https://www.uniprot.org/) for peptide identification and quantification. The FDR was maintained at 1% at the peptide-spectrum match, peptide, and protein levels. Differentially expressed proteins (DEPs) were defined as those with a |log_2_ FC| >1.5 and FDR < 0.05. For integrative analysis, DEGs and DEPs were cross-compared to identify overlapping molecules with concordant expression patterns at the transcriptomic and proteomic levels. Functional enrichment analyses were then conducted on the combined DEG and DEP datasets to determine commonly enriched pathways and biological processes.

The transcriptomic sequencing dataset produced in this study has been submitted to the NCBI BioProject database under accession number PRJNA1459301 (https://www.ncbi.nlm.nih.gov/bioproject/PRJNA1459301). The mass spectrometry-based proteomics dataset has been deposited in the ProteomeXchange Consortium through the PRIDE partner repository with the dataset identifier PXD077847 (https://proteomecentral.proteomexchange.org/cgi/GetDataset?ID=PXD077847).

### 2.8 Animal Model

Following a 1-week acclimatization period, 20 female BALB/c nude mice aged 4–6 weeks and weighing 18–22 g were obtained from Hunan SJA Laboratory Animal Co., Ltd., Changsha, China. Before tumor-cell inoculation, the animals were randomly allocated to two groups: the oe-miR-NC group and the oe-miR-320d group (*n* = 10 per group). The mice were housed under specific pathogen-free conditions at 22°C ± 2°C and 50% ± 10% relative humidity, with a 12-h light/dark schedule and free access to standard chow and water. S18 cells stably expressing oe-miR-NC or oe-miR-320d were collected, suspended in PBS, and adjusted to a density of 5 × 10^7^ cells/mL, For xenograft establishment, each mouse received a 100 μL subcutaneous injection of the cell suspension into the axillary region of the right forelimb. Tumor formation was considered successful once a palpable mass became visible and reached approximately 5 mm in diameter. During the experiment period, the mice were examined daily for general health, behavior, body weight, and tumor growth. Tumor length and width were measured with calipers, and tumor volume was calculated using the formula: volume = (length × width^2^)/2. The animals were euthanized after 3 weeks or when the longest tumor diameter approached 2 cm, without exceeding this limit, in accordance with humane endpoints.

All procedures were conducted following the ARRIVE guidelines and were approved by the Animal Ethics Committee of Hunan Normal University (Approval No. 797/2025). All possible measures were taken to reduce animal distress and maintain experimental rigor.

### 2.9 Cell Coculture

HL-60 cells (ATCC, Manassas, VA, USA; catalog number: CCL-240) were routinely screened and confirmed to be free of mycoplasma contamination. Cells were authenticated using STR profiling according to the supplier’s guidelines. HL-60 cells were induced to differentiate into neutrophil-like cells (dHL-60) using 1 μM all-trans retinoic acid (ATRA; Sigma-Aldrich, Merck; catalog number: R2625) for 3 days.

Differentiation was assessed by cell morphology using Wright’s stain, following previously established and widely used protocols [20]. After differentiation, stable oe-miR-NC or oe-miR-320d S18 and 5-8F cells were directly cocultured with dHL-60 cells in the same culture wells at a ratio of 1:0.25 for 24 h. Cells were cocultured in RPMI-1640 medium supplemented with 10% heat-inactivated FBS, 100 U/mL penicillin, and 100 μg/mL streptomycin. No physical barrier was used between the two cell types. The coculture ratio of 1:0.25 was selected based on preliminary pilot experiments. After coculture, supernatants were collected for analysis of secreted factors by enzyme-linked immunosorbent assay (ELISA), while the mixed cell populations were harvested together for downstream protein assays.

### 2.10 ELISA

Supernatants from cocultured cells were centrifuged at 1000× *g* for 5 min at 4°C to remove cell debris and then stored at −80°C until analysis. IL-8 concentrations were determined with a commercial ELISA kit (Lianke Biotechnology Co., Ltd., Hangzhou, China; catalog number: LK-ELISA-008) according to the manufacturer’s protocol. Background correction was performed using a 570-nm reference wavelength. A standard curve was generated from serial dilutions of the supplied standards. Standards and samples were loaded into pre-coated wells, and the assay was completed strictly in according to the kit protocol. Absorbance at 450 nm was measured with a BioTek Epoch 2 microplate reader (BioTek Instruments, Inc., Winooski, VT, USA; version 2.0). Cytokine concentrations were derived from the standard curve in GraphPad Prism 9 software (GraphPad Software, San Diego, CA, USA; version 9.5.1) using a four-parameter logistic regression model. All samples were assayed in technical triplicate, and the mean value was used to calculate cytokine concentration.

### 2.11 Western Blotting Assay

Total protein was isolated from S18, 5-8F, and dHL-60 cells using Cell Lysis Buffer for Western or IP (Beyotime Biotechnology; catalog number: P0013), following the manufacturer’s instructions. Protein concentration was determined using a BCA Protein Assay Kit (Beyotime Biotechnology; catalog number: P0010) and absorbance was measured with a NanoDrop 2000 UV-Vis spectrophotometer (Thermo Fisher Scientific; Model: ND2000), according to the manufacturer’s instructions. Equal protein amounts (30 μg per lane) were boiled for denaturation, resolved by 10% SDS-PAGE, and transferred onto PVDF membranes for 4 h. Membranes were blocked with 5% nonfat milk for 1 h at room temperature (RT) and then incubated overnight at 4°C with primary antibodies against GP1BA (1:1000; Proteintech Group, Rosemont, IL, USA; catalog number: 12860-1-AP), FGG (1:1000; Proteintech Group; catalog number: 15841-1-AP), HDAC10 (1:1000; Proteintech Group; catalog number: 19538-1-AP), GAPDH (1:1000; Proteintech Group; catalog number: 12457-1-AP), PADI4 (1:1000; Proteintech Group; catalog number: 17373-1-AP), MPO (1:1000; Proteintech Group; catalog number: 22225-1-AP), NE (1:500; Proteintech Group; catalog number: 27642-1-AP), NF-κB p65 (1:1000; Proteintech Group; catalog number: 80979-1-RR), and phospho-NF-κB p65 (1:1000; Proteintech Group; catalog number: 82335-1-RR). After three washes with Tris-buffered saline containing 0.1% Tween-20, membranes were incubated in the dark for 1 h at RT with fluorescent secondary antibodies, including goat anti-mouse IgG (1:1000; LI-COR Biosciences, Lincoln, NE, USA; catalog number: D00115-03) and goat anti-rabbit IgG (1:1000; LI-COR Biosciences; catalog number: C70426-05). Protein bands were detected using an Odyssey infrared imaging system (LI-COR Biosciences; version 3.0) and quantified with ImageJ software (NIH; Version 1.53c). Each experiment was independently repeated three times.

### 2.12 Immunohistochemical Assay

Tumor tissues from each group were fixed in 10% neutral buffered formalin (Biosharp, Hefei, China; catalog number: BL539A) for 24 h at RT and sectioned at 4 μm using a microtome. Sections were deparaffinized in xylene (Sinopharm Chemical Reagent Co., Ltd., Shanghai, China; catalog number: 10023418) and rehydrated through a graded ethanol solutions (Sinopharm Chemical Reagent Co., Ltd.). Antigen retrieval was then performed in citrate buffer (Beyotime Biotechnology; catalog number: P0083; pH 6.0) by microwave heating for 15 min. Sections were then blocked with 10% goat serum (Boster Biological Technology Co., Ltd., Wuhan, China; catalog number: AR0009) for 30 min at RT and subsequently incubated overnight at 4°C with primary antibodies against GP1BA (1:50, Proteintech Group; catalog number: 12860-1-AP), FGG (1:200, Proteintech Group; catalog number: 15841-1-AP), and HDAC10 (1:200, Proteintech Group; catalog number: 19538-1-AP). After washing, the sections were incubated with the corresponding HRP-conjugated secondary antibodies (1:200, Boster Biological Technology Co., Ltd.; catalog number: BA1054) for 30 min at RT (20–25°C). Immunoreactivity was visualized using diaminobenzidine (Solarbio, catalog number: DA1010) for 3–5 min, followed by counterstaining with hematoxylin (Solarbio, catalog number: G1120). After dehydration, clearing, and mounting with neutral resin mounting medium (Solarbio, catalog number: G8590), the stained sections were observed and photographed under a light microscope (Olympus Corporation; Model: BX53). The integrated optical density of positive staining was quantified using ImageJ software (NIH; Version 1.53c). 

### 2.13 Immunofluorescence Assay

Tumor tissues from each group were fixed in 10% neutral buffered formalin (Biosharp, catalog number: BL539A) for 24 h at RT and sectioned at 4 μm using a microtome. Sections were deparaffinized in xylene (Sinopharm Chemical Reagent Co., Ltd.; catalog number: 10023418), rehydrated through a graded ethanol solutions (Sinopharm Chemical Reagent Co., Ltd.; catalog number: 10009218), and subjected to antigen retrieval in citrate buffer (Beyotime Biotechnology; catalog number: P0083; pH 6.0) by microwave heating for 15 min. After blocking with 10% goat serum (Boster Biological Technology Co., Ltd., catalog number: AR0009) for 30 min at RT, sections were then incubated overnight at 4°C with primary antibodies against PADI4 (1:200; Proteintech Group; catalog number: 17373-1-AP), MPO (1:50; Proteintech Group; catalog number: 22225-1-AP), NE (1:200; Proteintech Group; catalog number: 27642-1-AP), and LY6G (1:200; Proteintech Group; catalog number: 65078-1-lg). For double immunofluorescence staining, two primary antibodies raised in different species were applied in combination as appropriate. After washing with PBS, the sections were incubated in the dark with fluorophore-conjugated secondary antibodies, including Alexa Fluor 488-conjugated goat anti-rabbit IgG (1:500; Invitrogen, Carlsbad, CA, USA; catalog number: A-11008) and Alexa Fluor 594-conjugated goat anti-mouse IgG (1:500, Invitrogen; catalog number: A-11005), for 1 h at RT. The nuclei were counterstained with DAPI-containing mounting medium (Vector Laboratories, Newark, CA, USA; catalog number: H-1200). Fluorescence images were acquired using a fluorescence microscope (Olympus Corporation; Model: Olympus IX73), and fluorescence intensity and colocalization were quantified using ImageJ software (NIH; Version 1.53c).

### 2.14 Statistical Analysis

Statistical analysis was conducted using GraphPad Prism 8.0 software (GraphPad Software, Inc.; version 8.0.0). Data are expressed as the mean ± standard deviation (SD). Each experiment was independently repeated three times. Comparisons between two groups were analyzed using an unpaired Student’s *t* test, whereas comparisons involving more than two groups were assessed by one-way analysis of variance (ANOVA) followed by Tukey’s multiple comparisons test. A *p*-value of <0.05 was considered statistically significant.

## 3 Results

### 3.1 Verification of Stable miR-320d Overexpression Efficiency

**Figure 1:**
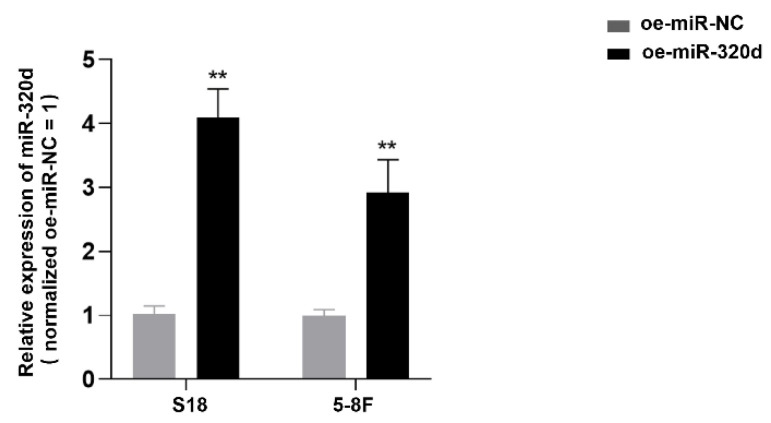
**qRT-PCR analysis of miR-320d overexpression.** Relative miR-320d expression levels in S18 and 5-8F cells transfected with oe-miR-320d or oe-miR-NC. U6 served as the internal control. Data are presented as mean ± SD (*n* = 3). **p < 0.01, compared to the oe-miR-NC group.

To investigate whether oe-miR-NC and oe-miR-320d were successfully transduced into NPC cells, the miR-320d expression level in NPC cells was quantified. qRT-PCR results showed that, compared to the oe-miR-NC group, the oe-miR-320d group exhibited a significant increase in miR-320d expression after the stable cell lines had been established and cultured for 24 h (Fig. 1), which was consistent with the expected outcome.

### 3.2 miR-320d Suppresses the Viability, Migration, and Invasion of NPC Cells

**Figure 2:**
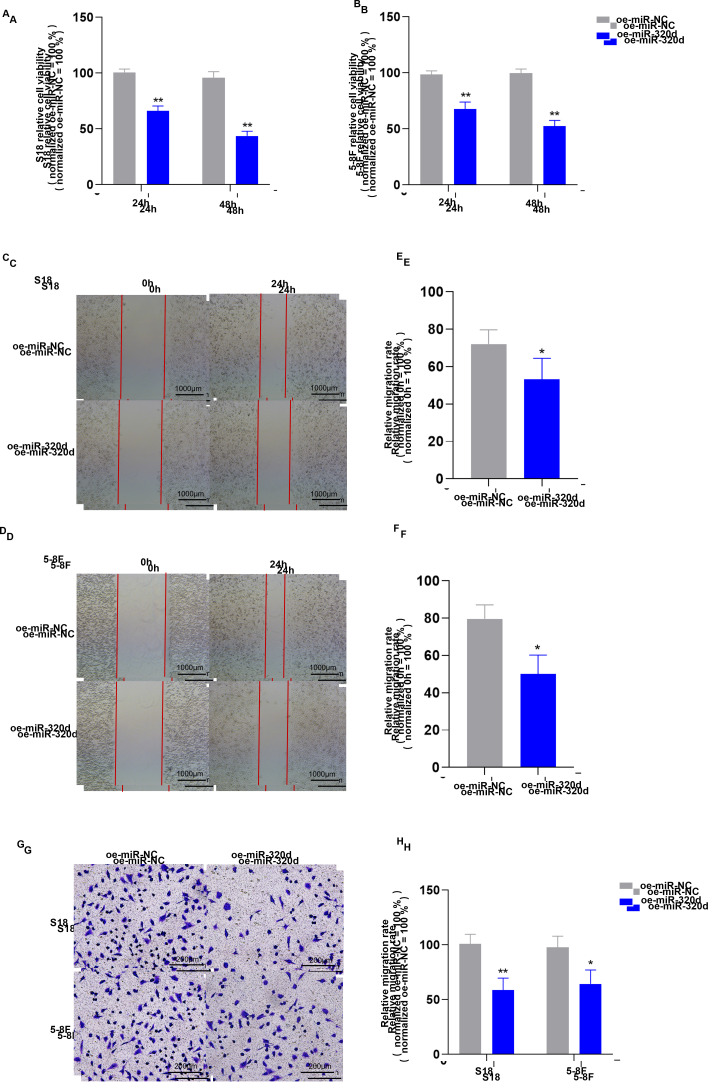
**miR-320d inhibits the viability, migration, and invasion abilities of S18 and 5-8F cells.** (**A**) CCK-8 assay results show that overexpression of miR-320d significantly reduced the relative survival rate of S18 cells. (**B**) CCK-8 assay results show that miR-320d overexpression significantly reduced the relative survival rate of 5-8F cells. (**C**) Scratch assay demonstrates that miR-320d overexpression inhibited the migration ability of S18 cells. (**D**) Scratch assay demonstrates that miR-320d overexpression inhibited the migration ability of 5-8F cells. (**E**) Quantitative analysis of S18 cell migration rate in the scratch assay. (**F**) Quantitative analysis of 5-8F cell migration rate in the scratch assay. (**G**) The Transwell invasion assay shows that miR-320d overexpression inhibited the invasion ability of S18 and 5-8F cells, with representative crystal violet staining images shown. (**H**) Quantitative analysis of the number of cells that migrated through the membrane in the invasion assay. Data are presented as mean ± SD (*n* = 3). *p < 0.05, **p < 0.01, compared to the oe-miR-NC group.

To define the biological function of miR-320d in NPC cells, we assessed cell viability, migration, and invasion. CCK-8 assay results demonstrated that, compared to the oe-miR-NC group, the oe-miR-320d group showed significantly reduced cell viability at both 24 h and 48 h (Fig. 2A,B); this finding suggests that miR-320d overexpression inhibits NPC cell viability. Wound healing assay results demonstrated that the wound closure rate was significantly slower in the oe-miR-320d group compared to that in the oe-miR-NC group (Fig. 2C,D). Quantitative analysis further indicated that the relative migration rate of the oe-miR-320d group was significantly lower than that of the oe-miR-NC group (Fig. 2E,F), suggesting that miR-320d overexpression suppresses the migration ability of NPC cells.

Transwell invasion assays revealed that the number of cells that migrated through the membrane was markedly reduced in the oe-miR-320d group compared to that in the oe-miR-NC group (Fig. 2G). Quantitative results further confirmed that miR-320d overexpression significantly inhibits the invasion ability of NPC cells (Fig. 2H).

In conclusion, miR-320d overexpression significantly inhibits the viability, migration, and invasion of NPC cells, indicating that miR-320d may play a potential tumor-suppressive role in NPC development.

### 3.3 Sequencing Result Analysis

**Figure 3:**
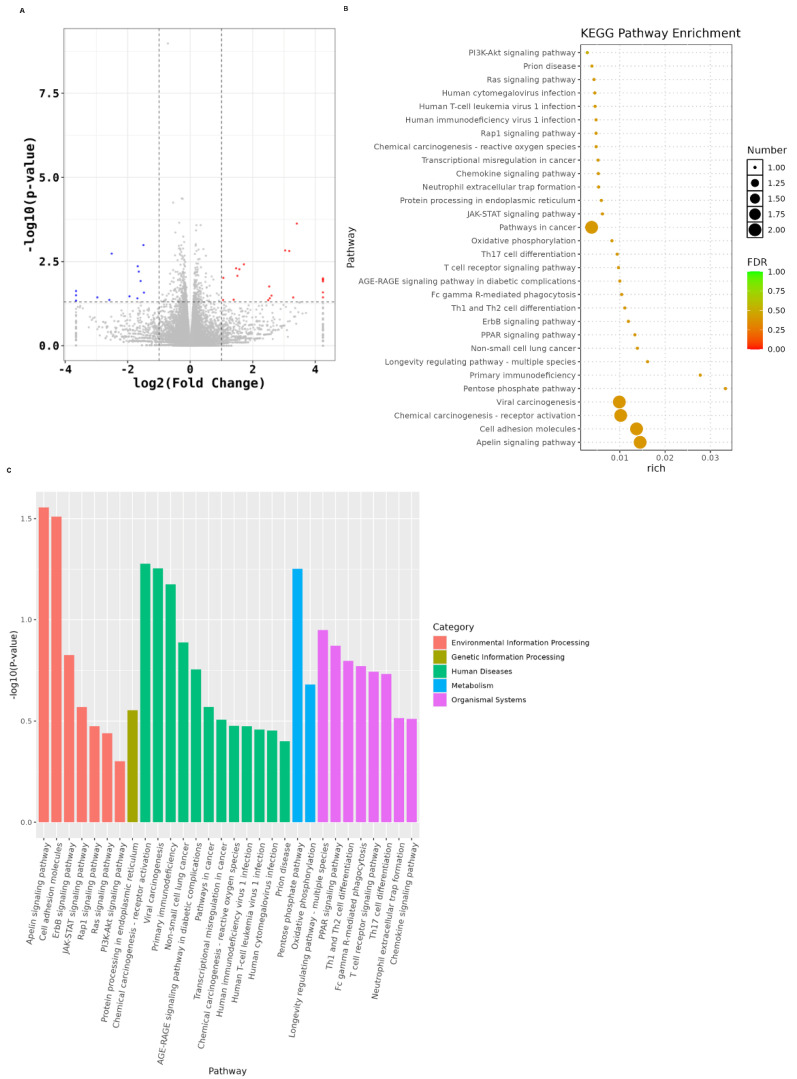
**Transcriptomic analysis.** (**A**) Volcano plot: The *x*-axis represents log2 Fold Change (FC), and the *y*-axis represents −log_10_ (*p*-value). (**B**) KEGG pathway enrichment bubble plot: The *y*-axis represents the pathway name, and the *x*-axis represents the enrichment ratio (rich factor). (**C**) KEGG pathway classification bar plot of DEGs: Pathways are categorized into five major groups: Environmental Information Processing, Genetic Information Processing, Human Diseases, Metabolism, and Organismal Systems. The plot shows the top enriched pathways and their −log_10_ (*p* value).

To further investigate the potential molecular mechanisms of miR-320d in NPC cells, we conducted a combined transcriptomic and proteomic analysis on cell samples from the oe-miR-NC and oe-miR-320d groups of S18 cells. Transcriptomic analysis, using predefined thresholds (|log_2_FC| ≥ 1, FDR < 0.05), identified 34 differentially expressed transcription factors (DETFs), with 21 upregulated and 13 downregulated TFs (Fig. 3A). KEGG functional enrichment analysis revealed that these DETFs were primarily involved in signal transduction, metabolic regulation, and disease-related pathways (Fig. 3B,C).

**Figure 4:**
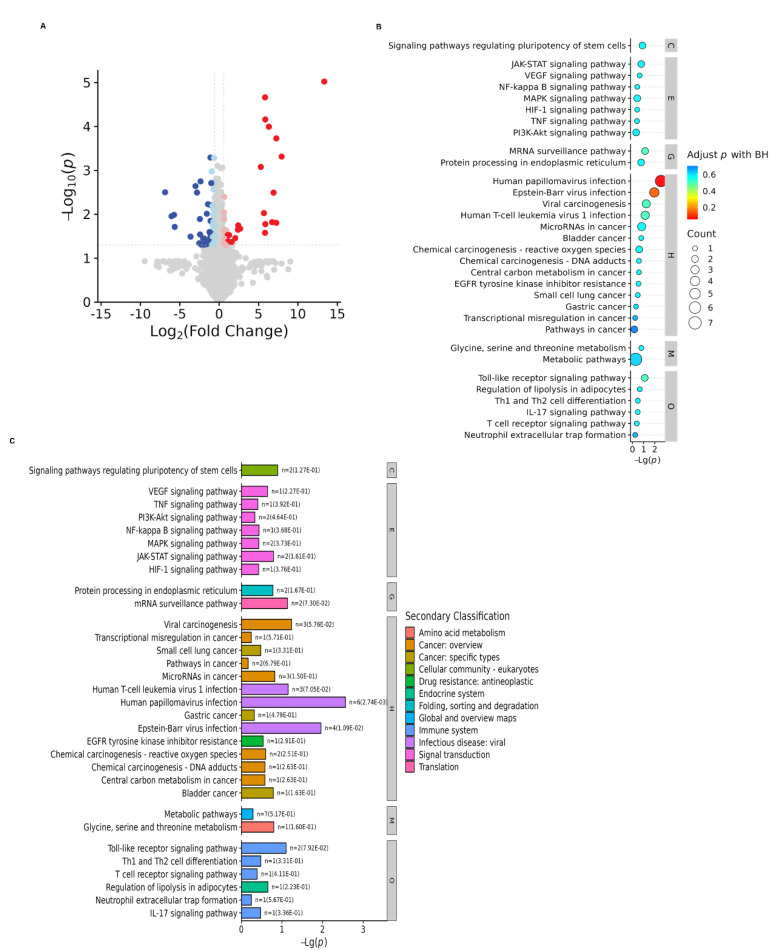
**Proteomic analysis.** (**A**) Volcano plot: The *x*-axis represents the log-transformed FC, and the *y*-axis represents the negative log-transformed *p*-value. (**B**) KEGG pathway enrichment bubble plot: The *x*-axis represents −log_10_ (*p*-value), and the *y*-axis represents significantly enriched pathway names. (**C**) Bar plot of KEGG pathways enriched with DEPs: The plot shows the top enriched pathways and their −log_10_ (*p*-value). Different colors represent the secondary functional classifications of pathways, including metabolism, signal transduction, the immune system, viral infections, and other biological processes.

Proteomic analysis, using a threshold of |log_2_FC| > 1.5 and FDR < 0.05, identified 88 DEPs, with 51 upregulated and 37 downregulated proteins (Fig. 4A). KEGG enrichment analyses showed that these DEPs were significantly enriched in biological processes and pathways related to metabolism, signal transduction, immune response, and viral infection (Fig. 4B,C).

**Figure 5:**
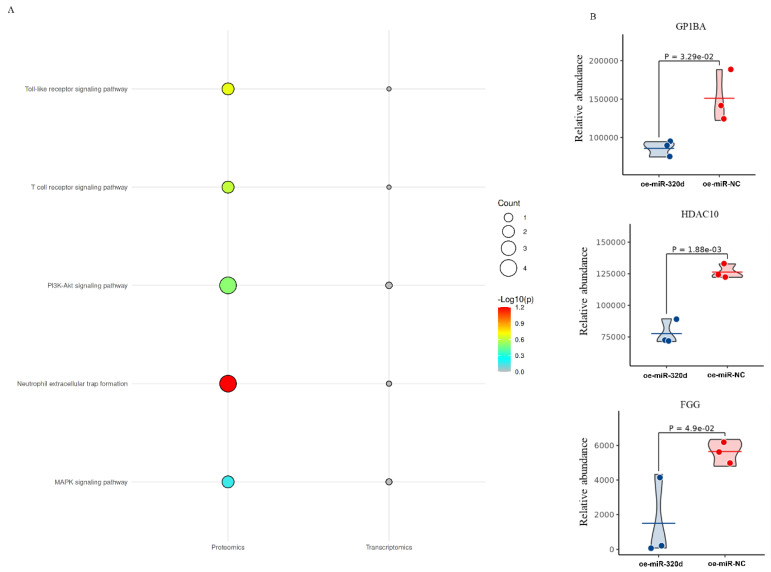
**Shared KEGG pathway analysis of transcriptomics and proteomics.** (**A**) Representative overlapping tumor and immune related KEGG candidate pathways prioritized according to Rich Factor and biological relevance are shown. The *x*-axis represents the groups (Proteomics and Transcriptomics), and the *y*-axis represents the selected KEGG pathway names. (**B**) Differences in the expression of key proteins GP1BA, HDAC10, and FGG in the NETs pathway between the oe-miR-320d and oe-miR-NC groups. Data are presented as relative abundance, with the *x*-axis representing the treatment groups and the *y*-axis representing protein expression levels. *p*-values were calculated using a *t*-test, with significance indicated as shown in the figure.

To integrate the two datasets at the pathway level, we compared the KEGG pathway results from the transcriptomic and proteomic analyses. Overlapping tumor- and immune-related KEGG pathways were then prioritized according to Rich Factor and biological relevance, including NETs, Toll-like receptor signaling pathway, T cell receptor signaling pathway, PI3K-AKT signaling pathway, and MAPK signaling pathway (Fig. 5A, Table 1). Among these pathways, NETs showed a relatively high Rich Factor and closer relevance to neutrophil-mediated immune regulation. Therefore, NETs were selected for subsequent validation. Candidate molecules for validation were selected from DEPs mapped to these overlapping pathways, because proteins are direct functional effectors of cellular phenotypes. Proteomic results further showed that proteins associated with the NETs pathway, including GP1BA, HDAC10, and FGG, were all downregulated in the oe-miR-320d group (Fig. 5B). Subsequent *in vivo* and *in vitro* experiments were performed to validate whether miR-320d participates in NPC progression by inhibiting the formation of NETs.

**Table 1:** Selected overlapping tumor and immune related KEGG candidate pathways and corresponding DEPs prioritized according to Rich Factor and biological relevance.

Pathway Name	Rich Factor	Symbols
Toll−like receptor signaling pathway	2.36	IRF, MAP3K8
T cell receptor signaling pathway	2.1	PPP2R3B, MAP3K8
PI3K−AKT signaling pathway	1.42	COL1A2, PPP2R3B, ITGB, FGFR3
Neutrophil extracellular trap formation	2.66	GP1BA, HDAC10, FGG
MAPK signaling pathway	0.85	FGFR, MAP3K8

### 3.4 miR-320d Downregulates the Expression of NETs Pathway-Related Proteins in NPC Cells

**Figure 6:**
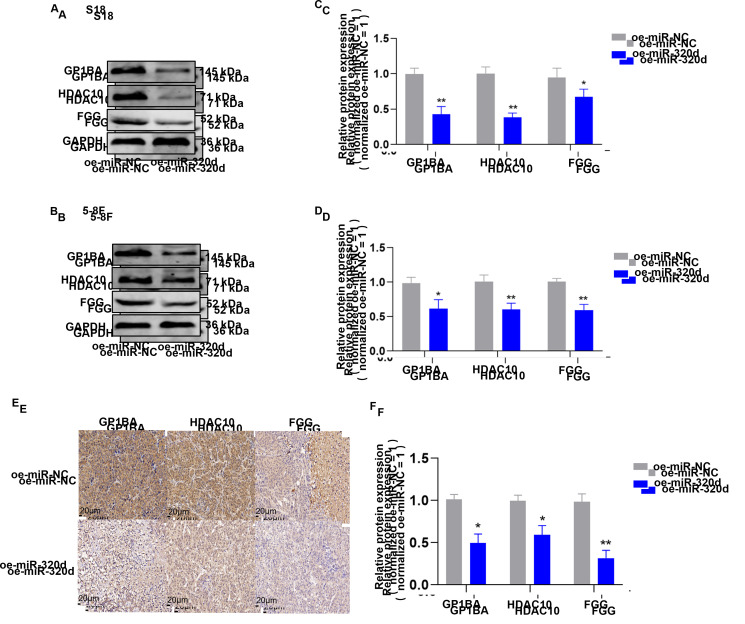
**miR-320d inhibits the expression of NETs pathway-related proteins in S18 and 5-8F cells and xenograft tumor tissues.** (**A**) Western blotting analysis results show that miR-320d overexpression downregulates the expression of GP1BA, HDAC10, and FGG proteins in S18 cells. (**B**) Quantitative analysis of the protein grayscale values corresponding to (A). (C) Western blotting analysis results show that miR-320d overexpression downregulates the expression of GP1BA, HDAC10, and FGG proteins in 5-8F cells. (**D**) Quantitative analysis of the protein grayscale values corresponding to (**C**). (**E**) Immunohistochemical staining results show that miR-320d overexpression reduces the expression of GP1BA, HDAC10, and FGG proteins in nude mouse xenograft tumor tissues. (**F**) Semi-quantitative analysis of the immunohistochemical analysis results corresponding to (**E**). Data are presented as mean ± SD (*n* = 3). *p < 0.05, **p < 0.01, compared to the oe-miR-NC group.

To validate the sequencing results, we examined the expression levels of NETs pathway-related proteins GP1BA, HDAC10, and FGG in NPC cells from the oe-miR-NC and oe-miR-320d groups. Western blotting analysis revealed that miR-320d overexpression significantly downregulated the expression levels of GP1BA, HDAC10, and FGG proteins in the S18 cell line, with significant differences compared to the oe-miR-NC group (Fig. 6A,B). In 5-8F cells, miR-320d overexpression also significantly reduced the expression of GP1BA, HDAC10, and FGG proteins (Fig. 6C,D).

Immunohistochemical assay showed that, compared to the OE-miR-NC group, the intensity of positive staining for GP1BA, HDAC10, and FGG was significantly weaker in the tumor tissues from the oe-miR-320d group. Quantitative analysis further confirmed the significant decrease in protein expression levels (Fig. 6E,F). These results were consistent with the proteomic sequencing data and suggest that miR-320d may regulate the formation of NETs by downregulating the expression of GP1BA, HDAC10, and FGG, thereby participating in processes related to NPC progression.

### 3.5 miR-320d Downregulates the Expression of NETs-Associated Proteins in Neutrophil-Like dHL-60 Cells

**Figure 7:**
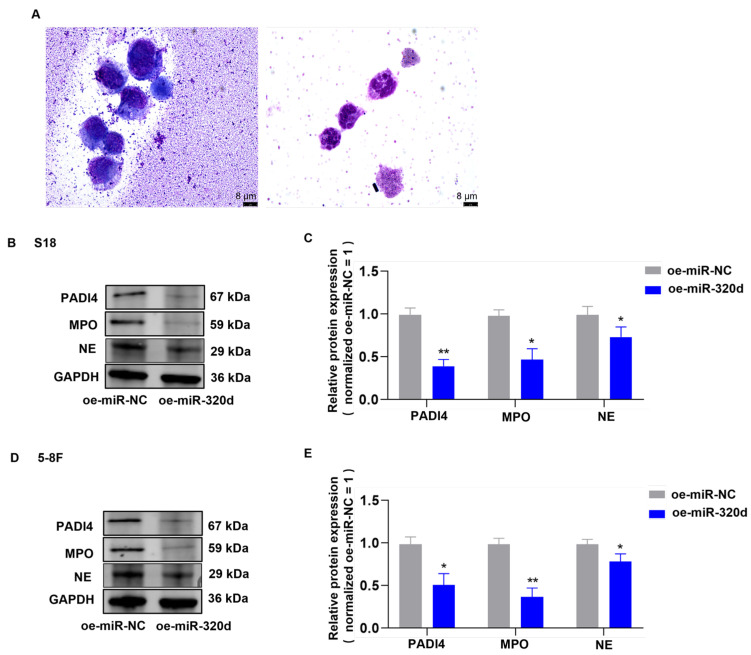
**miR-320d inhibits the expression of NETs-related proteins in dHL-60 cells.** (**A**) Morphological observation of ATRA induced differentiation of human HL-60 cells into neutrophil-like cells. The left image shows untreated HL-60 cells, whereas the right image shows ATRA-induced dHL-60 cells. Compared with untreated HL-60 cells, dHL-60 cells exhibited neutrophil-like morphological changes, including increased nuclear lobulation. (**B**) Western blotting analysis results show that miR-320d overexpression downregulates the expression of PADI4, MPO, and NE proteins in dHL-60 cells co-cultured with S18 cells. (**C**) Quantitative analysis of the protein expression corresponding to (**B**). (**D**) Western blotting analysis results show that miR-320d overexpression downregulates the expression of PADI4, MPO, and NE proteins in dHL-60 cells co-cultured with 5-8F cells. (**E**) Quantitative analysis of the protein expression corresponding to (**D**). Data are presented as mean ± SD (*n* = 3). *p < 0.05, **p < 0.01, compared to the oe-miR-NC group.

To simulate neutrophil infiltration in the tumor microenvironment, we induced human promyelocytic leukemia cells (HL-60) to differentiate into neutrophil-like dHL-60 cells using 1 μM ATRA. Morphological observations revealed that the induced dHL-60 cells exhibited a reduction in cell size (with a decrease in diameter from 14.38 μm to 12.17 μm), and the nuclei displayed a characteristic irregularly lobulated or granular shape, confirming successful differentiation (Fig. 7A). Next, we co-cultured dHL-60 cells with NPC cells from different groups to investigate the paracrine regulation of miR-320d on NETs formation. To assess whether miR-320d is involved in the formation of NETs in neutrophils, we analyzed the expression of NETs-associated proteins, including PADI4, MPO, and NE, in dHL-60 cells from the oe-miR-NC and oe-miR-320d groups. Western blotting analysis showed that the expression of these proteins, namely PADI4, MPO, and NE, was significantly reduced in dHL-60 cells from the oe-miR-320d group compared to those in the oe-miR-NC group (Fig. 7B,C). Similar results were observed in co-cultures with 5-8F cells, where miR-320d overexpression significantly inhibited the expression of PADI4, MPO, and NE in dHL-60 cells (Fig. 7D,E).

**Figure 8:**
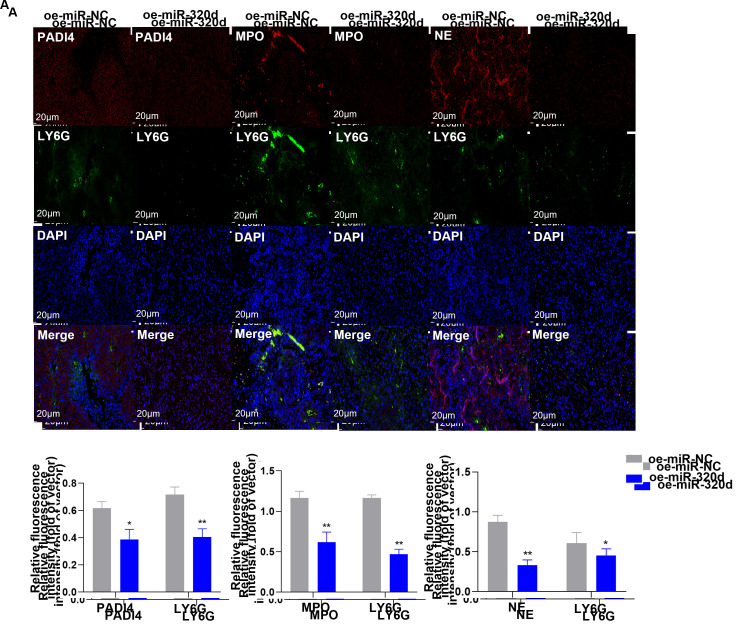
**miR-320d inhibits the expression of NETs-related proteins in nude mouse xenograft tumor tissues.** (**A**) Immunofluorescence co-staining results show that miR-320d overexpression downregulates the expression of PADI4, MPO, and NE (red) and neutrophil marker LY6G (green) in nude mouse xenograft tumor tissues. (**B**) Quantitative analysis of the fluorescence intensity corresponding to (**A**). Data are presented as mean ± SD (*n* = 3). *p < 0.05, **p < 0.01, compared to the oe-miR-NC group.

Furthermore, to further confirm the effect of miR-320d on NETs-associated proteins *in vivo*, we conducted an immunofluorescence assay. The results showed that, compared to the oe-miR-NC group, the intensity of positive staining for PADI4, MPO, and NE was markedly decreased in tumor tissues from the oe-miR-320d group. Quantitative immunofluorescence assay further confirmed a significant reduction in the relative fluorescence intensity of these proteins (Fig. 8A,B).

In conclusion, miR-320d significantly downregulates the expression of NETs-related proteins PADI4, MPO, and NE in neutrophils, suggesting that it may play a crucial role in NPC tumor progression.

### 3.6 miR-320d Downregulates the Expression of Proteins in the NF-κB/IL-8 Signaling Pathway

**Figure 9:**
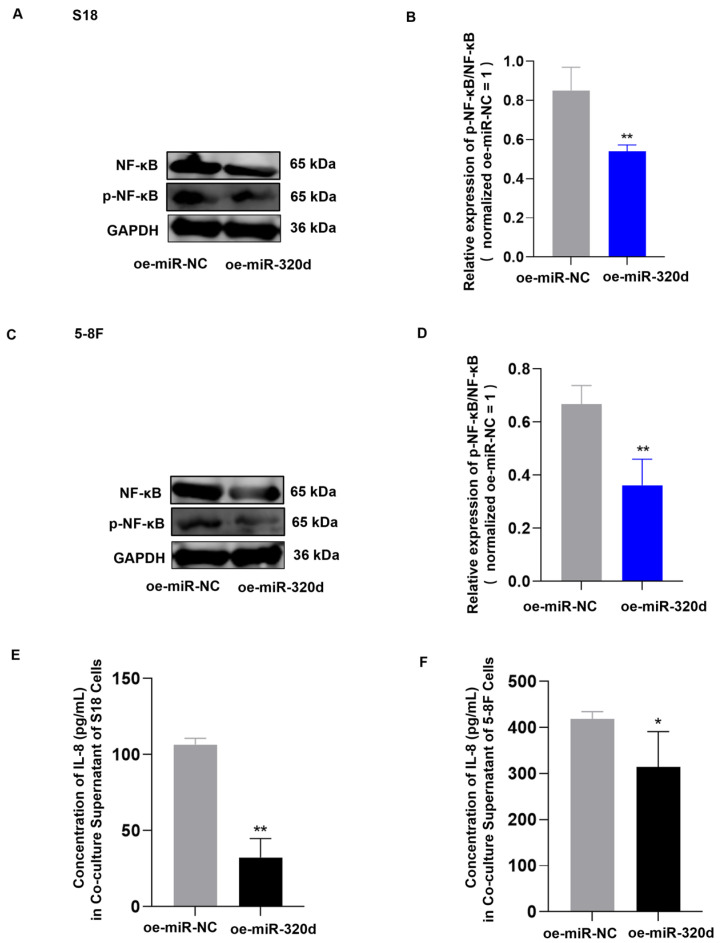
**miR-320d inhibits the NF-κB/IL-8 signaling pathway.** (**A**) Western blotting analysis demonstrates that miR-320d overexpression significantly reduces the p-NF-κB/NF-κB ratio in S18 cells. (**B**) Quantification of the protein levels corresponding to (**A**). (**C**) Western blotting analysis demonstrates that miR-320d overexpression significantly reduces the p-NF-κB/NF-κB ratio in 5-8F cells. (**D**) Quantification of the protein levels corresponding to (**C**). (**E**) ELISA results show that miR-320d overexpression reduces IL-8 levels in co-cultured medium with S18 cells. (**F**) ELISA results show that miR-320d overexpression reduces IL-8 in co-cultured medium with 5-8F cells. Data are presented as mean ± SD (*n* = 3). *p < 0.05, **p < 0.01, compared to the oe-miR-NC group.

To further elucidate the mechanism by which miR-320d inhibits NETs formation, we determined the expression of NF-κB signaling pathway-related proteins and IL-8 levels in the co-culture medium of NPC cells from the oe-miR-NC and oe-miR-320d groups. Western blotting analysis revealed a significant decrease in the p-NF-κB/NF-κB ratio in the oe-miR-320d group compared to that in the oe-miR-NC group in S18 cells (Fig. 9A,B). Similarly, a reduction in the p-NF-κB/NF-κB ratio was observed in 5-8F cells in the oe-miR-320d group relative to that in the oe-miR-NC group (Fig. 9C,D). ELISA results also indicated that the protein levels of IL-8 in the co-culture medium were significantly reduced in the oe-miR-320d group compared to those in the oe-miR-NC group (Fig. 9E,F). These findings suggest that miR-320d may play a regulatory role in NPC progression by inhibiting the NF-κB/IL-8 signaling pathway.

## 4 Discussion

Accumulating evidence indicates that miRNAs are critical regulators of NET formation and cancer-associated inflammatory responses. For example, miR-144 promotes NET-induced lung injury by inhibiting KLF2 and activating the NF-κB/CXCR1 signaling pathway [21]. Furthermore, the loss of miR-146a enhances NET formation, thereby aggravating systemic inflammation and increasing thrombotic risk in cancer [22], whereas miR-155 enhances NET formation by modulating PADI4 expression [23]. In the present study, KEGG enrichment analysis of DEGs and DEPs in S18 NPC cells between the oe-miR-NC and oe-miR-320d groups showed that miR-320d-regulated molecules were significantly enriched in NET-related pathways. Among these molecules, GP1BA, HDAC10, and FGG exhibited prominent changes in their expression, suggesting that miR-320d may be closely associated with the regulation of NET-related biological processes in NPC.

Previous studies have shown that GP1BA participates in inflammatory and immune regulation through interactions with receptors on monocytes and neutrophils and is involved in neutrophil activation and migration [24]. HDAC10 has also been implicated in neutrophil-associated immune regulation and inflammatory responses [25]. FGG, a major component of fibrinogen, contributes to neutrophil adhesion, migration, and inflammatory activation by participating in fibrinogen assembly and aggregation [26]. Collectively, these findings suggest that GP1BA, HDAC10, and FGG are functionally associated with NET formation and the inflammatory tumor microenvironment. In the present study, miR-320d overexpression significantly downregulated the expression of GP1BA, HDAC10, and FGG in NPC cells; this result was consistent with the proteomic sequencing results. These data support the notion that miR-320d may influence NET-related signaling networks in NPC.

MPO, PADI4, and NE are key effector molecules involved in NET formation. MPO promotes the generation of reactive oxygen species and is associated with tumor-promoting inflammation and poor prognosis in several malignancies [27,28]. PADI4 is a central enzyme in NET release because it catalyzes histone citrullination and facilitates chromatin decondensation; for example, IL-33 upregulates PADI4 and promotes NET formation in hepatocellular carcinoma [29]. NE contributes to chromatin decondensation during NET formation and mediates extracellular matrix degradation. Beyond its canonical role in NET biology, NE has also been implicated in tumor progression, immune remodeling, and EMT in multiple cancer types [30]. In the present study, miR-320d overexpression significantly reduced the expression of MPO, PADI4, and NE in dHL-60 cells. Therefore, miR-320d may attenuate NET formation in the tumor microenvironment by suppressing the expression of critical NET-associated molecules, thereby influencing NPC progression.

Accumulating evidence indicates that NETs play a critical role in tumor progression, metastasis, and therapeutic resistance. NETs not only facilitate tumor cell invasion and increase vascular permeability, thereby promoting cancer cell dissemination [31,32], but they also contribute to tumor angiogenesis and cancer-associated thrombosis, ultimately accelerating the growth and metastatic spread of multiple malignancies. The presence of NETs is frequently associated with poor prognosis and metastatic disease, and their targeted inhibition has been shown to be a promising therapeutic strategy in models such as breast cancer [33]. Further studies have demonstrated that aberrant NF-κB activation and NET formation may act synergistically to aggravate lung adenocarcinoma progression and reshape the tumor immune microenvironment [34]. Mechanistically, the NF-κB signaling pathway is a key regulator of IL-8 production. The activation of NF-κB promotes IL-8 secretion, which subsequently recruits neutrophils and enhances NET formation. Conversely, NETs can activate the TLR9/NF-κB pathway in macrophages, further stimulating IL-8 release and establishing an NF–κB–IL–8 positive feedback loop that amplifies inflammation and drives disease progression [35]. In pancreatic cancer, IL-8 induces NET formation through the CXCR1/2 signaling pathway, thereby mediating chemoresistance and protecting tumor cells from drug-induced death [36]. Therefore, targeting the NF-κB/IL-8 feedback loop has been proposed as a potential strategy to improve tumor sensitivity to therapy and alleviate NET-related inflammation [37].

In the present study, we found that miR-320d overexpression markedly suppressed the protein expression levels of NF-κB and IL-8 in NPC cells as well as in the supernatants of the co-culture system, suggesting that miR-320d exerts a tumor-suppressive role in NPC. However, the current data support an association between miR-320d overexpression and downregulation of the NF-κB/IL-8 axis rather than a direct causal mechanism. Therefore, the antitumor effect of miR-320d may, at least in part, involve modulation of the NF-κB/IL-8 pathway and inhibition of NET formation. These findings expand the current understanding of miRNA-mediated regulatory mechanisms in NPC and suggest that miR-320d not only affects the intrinsic biological behavior of tumor cells, but it may also participate in inflammation- and microenvironment-related regulatory networks [38,39]. Further mechanistic studies are required to determine whether the NF-κB/IL-8 axis directly mediates the antitumor effects of miR-320d in NPC. 

Although the miR-320 family functions as a tumor suppressor in multiple malignancies, miRNA-based therapy still faces substantial challenges, including poor stability, insufficient target specificity, limited delivery efficiency, off-target effects, and immune-related toxicity. Clinical studies of the liposomal miR-34a mimic MRX34 have shown the feasibility of miRNA therapeutics; however, they have also highlighted major limitations related to safety and delivery [40,41]. Therefore, while our findings support a mechanistic role for miR-320d in NPC, the current evidence is still insufficient to support its direct development as a therapeutic target. Rather, this study provides a rationale for further exploration of the translational value of modulating this axis or its downstream pathways.

Although directly targeting miR-320d is not yet clinically feasible, several downstream components of this pathway have been explored pharmacologically in other disease contexts. For example, CXCR1/2 antagonists such as reparixin can inhibit IL-8 signaling and exhibit antitumor activity [42], whereas PAD4 inhibitors, including Cl-amidine and BMS-P5, can suppress NETosis [43,44]. However, the therapeutic relevance of these agents in NPC remains largely unexplored. Therefore, the pathway identified in the present study provides a mechanistic basis for future investigations, rather than direct evidence for immediate clinical translation in patients with NPC.

This study has several limitations. First, it remains to be validated whether GP1BA, HDAC10, and FGG are the direct target genes of miR-320d. Additionally, it remains unclear whether the inhibitory effect of miR-320d on the NET pathway is direct or indirect. Second, the current conclusions are based primarily on *in vitro* experiments and animal models; thus, the actual role of this axis in the human NPC microenvironment and its association with clinical outcomes require validation in large-scale clinical studies. Moreover, this study did not examine the relationship between miR-320d expression and inflammation- or NET-related biomarkers in clinical samples. Future studies integrating animal models, clinical cohorts, and interventional studies targeting key nodes of this axis are warranted to further clarify its clinical significance.

## 5 Conclusions

This study demonstrates that miR-320d exerts a tumor-suppressive effect in NPC and that this effect may be associated, at least in part, with modulation of the NF-κB/IL-8 signaling axis and reduced NETs. These findings expand our current understanding of miRNA-mediated inflammatory and microenvironmental regulation in NPC progression and suggest that the miR-320d/NF-κB/IL-8/NET pathway may represent a potentially important regulatory network. Although the direct mechanistic role of this pathway requires further investigations, the present study provides a basis for future exploration of its potential translational relevance and that of its downstream effectors.

## Data Availability

The datasets used and/or analyzed during the current study are available from the corresponding author upon reasonable request.
